# Changes in mortality disparities by education in Russia from 1998 to 2017: evidence from indirect estimation

**DOI:** 10.1093/eurpub/ckab070

**Published:** 2021-05-02

**Authors:** Vladimir M Shkolnikov, Evgeny M Andreev, Domantas Jasilionis

**Affiliations:** 1 Laboratory of Demographic Data, Max Planck Institute for Demographic Research, Rostock, Germany; 2 International Laboratory for Population and Health, National Research University Higher School of Economics, Moscow, Russia

## Abstract

This article addresses two unresolved methodological issues related to prior research on Russia that was based on census-unlinked data and did not account for the substantial increase in the share of death records with missing information on education. The study uses a proportional mortality analysis method relying on a case–control framework, together with a plausible imputation-based solution for the redistribution of the unknown education on death records. The new results suggest that high levels of inequality persist, but they do not support recent findings indicating that the educational gap in life expectancy has substantially widened.

## Introduction

In response to the recent marked improvements in life expectancy in Russia, questions have been raised about the extent to which this progress has been equitable across socioeconomic groups. Prior studies on Russia based on unlinked mortality data have reported that mortality disparities by education widened considerably during the 1990s.^1^^,^^2^ The first two studies documenting the trends in life expectancy disparities after 1998 in Russia were published following the reintroduction of the practice of providing information on education on death records in 2011. These studies reported that there was a further widening of mortality differentials between 1998 and 2015.^3^^,^^4^ Unfortunately, this new evidence was based on problematic census-unlinked data drawn from two different and independent sources of information on education for deaths and population exposures. It is well known that a mismatch in the source of information can lead to numerator–denominator bias in group-specific death rates.^5–7^ Besides, neither of these studies properly accounted for the substantial increase in the share of death records with missing information on education. In the current study, we address these two unresolved methodological challenges and propose alternative and adjusted estimations of the levels and the changes in mortality differentials by education in Russia from 1998 to 2017. 

## Methods

The study uses anonymous vital registration death records by age, sex, cause of death and education in Russia for the years 1998 and 2017 that were provided by the Russian State Statistical Service (Rosstat). Death records with unknown age (0.08%), records with age at death below 30 (3.9%) and above 80 (16.7%) were excluded. To improve the comparability of the records across time, only three broad aggregated educational categories referring to the highest completed education were used: higher education (university or other higher education), secondary education (including upper secondary, professional secondary and incomplete higher education) and lower than secondary education (incomplete secondary and lower education). The share of death records with missing information on education was 6.3% in 1998 and 23.7% in 2017. Total numbers and distributions of deaths according to the original and estimated education used for the analyses are available in Supplementary table S1.

To avoid the numerator–denominator bias in mortality rates by education, we applied an indirect method based on proportional mortality analysis assuming no educational differences in mortality from leukemia.^8^ The method was implemented within the case–control study framework, with the cases being deaths from all causes but leukemia, and the controls being deaths from leukemia (ICD10 codes C91–C96). For each year and sex, the mortality impacts of the educational categories were estimated by the logistic model linking the dichotomous case–control variable with education and age (5-year age groups). To check whether the mortality impacts of education have changed, we ran additive (Education + Year) and interaction (Education × Year) logistic models on data including deaths in 1998 and 2017. While the outcomes of the interaction model allowed us to compare the mortality impacts of the same educational category between the 2 years, the post-estimation likelihood-ratio test indicated the statistical significance of the difference between the additive and the Education × Year models (Supplementary table S2).

To evaluate the influence of the missing education information on the results, we applied the statistical models to three types of data: (i) records with a separate unknown education category; (ii) records after exclusion of those with missing education and (iii) records with the unknown education filled in by imputed values. The latter estimates were obtained with the help of the multinomial logistic regression imputation method^9^ (see Supplementary Appendix S1 for further details).

## Results

Table 1 shows the logistic regression mortality odds ratios (ORs) by education calculated according to the three aforementioned statistical models. Generally, these models generate ORs that are quite similar: 2.3–2.6 in 1998 vs. 2.7–2.9 in 2017 for the lower education and 1.5–1.7 in 1998 vs. 1.6–1.8 in 2017 for secondary education. The confidence limits of the point estimates are overlapping between 1998 and 2017. The outcomes of the model using the unknown education as a separate category (Section A in table 1) reflect the changing composition of this group. In terms of excess mortality, the unknown category was in-between the lowest and the secondary education groups in 1998, whereas it was much closer to the secondary education group in 2017. These changes coincided with a more than tripling of the population weights of this category (Supplementary table S1).

**Table 1:** Mortality impacts from proportional mortality analysis expressed as logistic regression odds ratios^a^

	Males						Females					
	1998			2017			1998			2017		
	OR	95%CI		OR	95%CI		OR	95%CI		OR	95%CI	
(A) Estimates based on data with a separate unknown education category												
Unknown	2.033	1.702	2.427	1.679	1.511	1.865	1.858	1.544	2.237	1.468	1.316	1.637
Lower	2.565	2.289	2.874	2.959	2.537	3.450	2.479	2.183	2.814	2.925	2.514	3.403
Secondary	1.653	1.475	1.853	1.789	1.631	1.962	1.514	1.336	1.716	1.599	1.450	1.764
Higher	1 (ref.)			1 (ref.)			1 (ref.)			1 (ref.)		
(B) Estimates based on data with missing education excluded												
Lower	2.552	2.278	2.860	2.945	2.525	3.435	2.457	2.164	2.791	2.882	2.475	3.356
Secondary	1.665	1.485	1.866	1.792	1.633	1.966	1.517	1.338	1.719	1.600	1.451	1.765
Higher	1 (ref.)			1 (ref.)			1 (ref.)			1 (ref.)		
(C) Estimates based on data with imputed education^b^												
Lower	2.552^c^	2.278	2.860	2.849	2.480	3.272	2.327	2.050	2.642	2.722	2.385	3.107
Secondary	1.665	1.485	1.866	1.771	1.623	1.932	1.458	1.290	1.648	1.631	1.482	1.796
Higher	1 (ref.)			1 (ref.)			1 (ref.)			1 (ref.)		

Ref., reference category.

^a^Odds of dying from all causes except leukemia relative to odds of dying from leukemia by educational category.

^b^See ‘Methods’ for more details about the multiple imputations.

^c^In 1998 the odds ratios are the same in sections B) and C) of the table because the missing values of education in 1998 were excluded from the imputation procedure.

The interaction results presented in Supplementary table S2 further explore the statistical significance of the changes in relative mortality inequalities based on the same three modeling assumptions. During this step, we first tested whether the model with the interaction term between the year and education fit better than a simple model without the interaction (separately controlling for age, education and year). The results suggest that the models with the interaction term fit better only if the unknown education was used as a separate category mainly due to the diminishing mortality effect of the unknown education. For the remaining two solutions, no statistically significant changes in the education effects were found. These findings confirm that there were no statistically significant changes in the relative mortality differentials by education.

## Discussion

Monitoring socioeconomic mortality differentials remains a challenging task for Russia, due to the lack of census-linked longitudinal data, and the changes in the completeness of information on the educational attainment of the deceased. In this study, we proposed a plausible solution allowing to fix some of the limitations related to the unlinked mortality data. First, we applied a proportional mortality analysis method based on a case–control framework, which is free from numerator–denominator bias affecting the unlinked mortality data. Second, we proposed a plausible solution for the redistribution of the unknown education on death records, which became a much larger, more randomly distributed and less selective group in 2017 compared to 1998.

The key assumption of the proportional mortality method was that control cases (leukemia deaths) were independent of the education effect. Our decision to use leukemia was supported by the prior evidence from international and national studies suggesting the absence of an educational gradient in mortality from this cause of death.^10^ We would, however, warn that this approach should be applied as an alternative solution only in cases in which obtaining more reliable census-linked or register-based data is not possible.

Our study provides some insights into the directions of changes in mortality inequalities by education in Russia over two decades, from 1998 to 2017. While the findings based on the adjusted data and indirect methodology suggest that the level of inequality remains very high, they do not support recent evidence that there has been a substantial widening of the educational gap in life expectancy. These findings point to the importance of taking into account the missing category and distortions due to numerator–denominator bias in the unlinked data. Because persisting mortality inequalities may become an obstacle to the sustainability of overall health progress in Russia, greater efforts are needed to obtain a more reliable evidence base for monitoring this important public health issue.

## Supplementary data

Supplementary data are available at *EURPUB* online.

## Funding

This study has received funding from the Basic Research Program at the National Research University Higher School of Economics. The funder had no role in study design, data collection, and analysis, decision to publish, or preparation of the manuscript.

**Conflict of interests**

The authors declare that they have no conflict of interests.

**Key points**

- Prior studies reporting a substantial widening of the educational gap in life expectancy in Russia suffered from numerator–denominator bias and did not appropriately address the increase in the missing education cases.
- The current study proposes an indirect method that relies on a case–control framework and an imputation-based solution to overcome the methodological and data constraints.
- The new results highlight the persisting high mortality inequality, but they do not confirm the further large increase in inequalities.
- Greater efforts are needed to obtain a more reliable evidence base for monitoring mortality inequalities in Russia and many other countries in the Eastern European region.

## Supplementary Material

ckab070_Supplementary_Data
