# Supplementary material for: Changes in mortality disparities by education in Russia from 1998 to 2017: evidence from indirect estimation
Source: Eur J Public Health. 2021 May 2;32(1):21–3. doi: 10.1093/eurpub/ckab070 (PMC8807072; doi:10.1093/eurpub/ckab070)
Supplement: ckab070_Supplementary_Data [file ckab070_supplementary_data.zip › ejph-2020-12-sr-1524-File002.docx]

**Supplementary Table 1.** Percentage distributions of deaths due to causes other than leukemia and due to leukemia by education in 1998 and 2017 based on the original data and data with imputed education at ages 30 to 80

|  | 1998 | | | | 2017 | | | |
| --- | --- | --- | --- | --- | --- | --- | --- | --- |
|  | Original | | Imputed^b^ | | Original | | Imputed^b^ | |
|  | All but leukemia | Leukemia | All but  leukemia | Leukemia | All but leukemia | Leukemia | All but  leukemia | Leukemia |
|  | Males | | | | Males | | | |
| Lower | 53.3 | 42.2 | 53.3(53.1,53.3) | 42.2(40.4,44.1) | 16.0 | 8.9 | 20.3(20.2,20.4) | 12.2(11.0,13.5) |
| Secondary | 38.5 | 42.1 | 38.5(38.4,38.6) | 42.1(40.2,44.0) | 69.3 | 65.6 | 65.4(65.3,65.5) | 63.0(61.2,64.9) |
| Higher | 8.2 | 15.6 | 8.2(8.2,8.3) | 15.6(14.3,4,17.0) | 14.8 | 25.5 | 14.2(14.1,14.3) | 24.7(23.1,26.4) |
| N | 763086 | 2659 | 763086 | 2659 | 602484 | 2494 | 792692 | 2706 |
|  | Females | | | | Females | | | |
| Lower | 67.3 | 45.9 | 67.3(67.2,67.4) | 45.9(44.0,47.8) | 23.0 | 10.4 | 27.9(27.7,28.0) | 15.1(13.7,16.5) |
| Secondary | 27.5 | 42.4 | 27.5(27.4,27.6) | 42.4(40.5,44.3) | 64.2 | 67.8 | 59.9(59.8,60.0) | 63.8(61.9,65.5) |
| Higher | 5.2 | 11.7 | 5.2(5.2,5.3) | 11.7(10.5,12.9) | 12.9 | 21.8 | 12.2(12.1,12.3) | 21.2(19.6,22.7) |
| N | 637668 | 2706 | 637668 | 2706 | 473513 | 2454 | 617323 | 3283 |

Notes. ^b^ See “Methods” and Supplementary Annex for more details about the multiple imputation procedure.

**Supplementary Table 2.** Interaction results

| **A) Data with a separate unknown education category** | | | | | | | |
| --- | --- | --- | --- | --- | --- | --- | --- |
| \|  \| Males \| \| \| Females \| \| \| \| --- \| --- \| --- \| --- \| --- \| --- \| --- \| \|  \| OR^a^ \| 95%CI \| \| OR^a^ \| 95%CI \|  \| \| *Education* \|  \|  \|  \|  \|  \|  \| \| -Unknown \| 2.015 \| 1.688 \| 2.405 \| 1.931 \| 1.605 \| 2.323 \| \| -Lower \| 2.570 \| 2.295 \| 2.878 \| 2.729 \| 2.408 \| 3.093 \| \| -Secondary \| 1.631 \| 1.456 \| 1.827 \| 1.492 \| 1.316 \| 1.690 \| \| -Higher \| 1 (Ref.) \|  \|  \| 1 (Ref.) \|  \|  \| \| *Year* \|  \|  \|  \|  \|  \|  \| \| -2017 \| 0.944 \| 0.834 \| 1.069 \| 0.972 \| 0.845 \| 1.118 \| \| -1998 \| 1 (Ref.) \|  \|  \| 1 (Ref.) \|  \|  \| \| *Education*Year* \|  \|  \|  \|  \|  \|  \| \| -Unknown&Year=2017 \| 0.839 \| 0.683 \| 1.031 \| 0.755 \| 0.609 \| 0.936 \| \| -Lower&Year=2017 \| 1.147 \| 0.948 \| 1.388 \| 1.006 \| 0.828 \| 1.222 \| \| -Secondary&Year=2017 \| 1.106 \| 0.956 \| 1.280 \| 1.070 \| 0.913 \| 1.254 \| \| -Year=1998 \| 1 (Ref.) \|  \|  \| 1 (Ref.) \|  \|  \| \| *p* difference between the models: Year+Educ *vs*. Year*Educ \| 0.010 \| \| \| 0.002 \| \| \|   **B) Data with missing education excluded** | | | | | | | |
|  | Males | | | Females | | |  |
|  | OR^a^ | 95%CI | | OR^a^ | 95%CI | |  |
| *Education* |  |  |  |  |  |  |  |
| -Lower | 2.560 | 2.285 | 2.867 | 2.691 | 2.374 | 3.051 |  |
| -Secondary | 1.638 | 1.462 | 1.835 | 1.496 | 1.320 | 1.695 |  |
| -Higher | 1 (Ref.) |  |  | 1 (Ref.) |  |  |  |
| *Year* |  |  |  |  |  |  |  |
| -2017 | 0.939 | 0.829 | 1.063 | 0.964 | 0.838 | 1.109 |  |
| -1998 | 1 (Ref.) |  |  | 1 (Ref.) |  |  |  |
| *Education*Year* |  |  |  |  |  |  |  |
| -Lower&Year=2017 | 1.146 | 0.947 | 1.387 | 0.996 | 0.820 | 1.211 |  |
| -Secondary&Year=2017 | 1.107 | 0.957 | 1.281 | 1.067 | 0.910 | 1.250 |  |
| -Year=1998 | 1 (Ref.) |  |  | 1 (Ref.) |  |  |  |
| *p* difference between the models: Year+Educ *vs*. Year*Educ | 0.288 | | | 0.574 | | |  |

**C) Data with imputed education^b^**

|  | Males | | | Females | | |
| --- | --- | --- | --- | --- | --- | --- |
|  | OR^a^ | 95%CI | | OR^a^ | 95%CI | |
| *Education* |  |  |  |  |  |  |
| -Lower | 2.567 | 2.292 | 2.875 | 2.735 | 2.413 | 3.100 |
| -Secondary | 1.633 | 1.458 | 1.830 | 1.491 | 1.316 | 1.690 |
| -Higher | 1 (Ref.) |  |  | 1 (Ref.) |  |  |
| *Year* |  |  |  |  |  |  |
| -2017 | 0.931 | 0.825 | 1.051 | 0.918 | 0.800 | 1.053 |
| -1998 | 1 (Ref.) |  |  | 1 (Ref.) |  |  |
| *Education*Year* |  |  |  |  |  |  |
| -Lower&Year=2017 | 1.106 | 0.925 | 1.323 | 1.009 | 0.838 | 1.216 |
| -Secondary&Year=2017 | 1.095 | 0.950 | 1.263 | 1.097 | 0.937 | 1.284 |
| -Year=1998 | 1 (Ref.) |  |  | 1 (Ref.) |  |  |

Notes. ^a^ Odds of dying from all causes except leukemia relative to odds of dying from leukemia by educational category. ^b^ See “Methods” and Supplementary Annex for more details about the multiple imputation procedure. Ref. – reference category.
