# Supplementary material for: Changes in mortality disparities by education in Russia from 1998 to 2017: evidence from indirect estimation
Source: Eur J Public Health. 2021 May 2;32(1):21–3. doi: 10.1093/eurpub/ckab070 (PMC8807072; doi:10.1093/eurpub/ckab070)
Supplement: ckab070_Supplementary_Data [file ckab070_supplementary_data.zip › ejph-2020-12-sr-1524-File003.docx]

**Supplementary Appendix S1**

**Imputation of missing education**

Information about the educational attainment of the deceased originated from anonymous death records of 1998 and 2017 provided by Rosstat.^s1^ The practice of mandatory registration of education in death records in Russia stopped in 2002-10 and resumed in 2011. However, the completeness of information about the education of the deceased in 2017 greatly varied across the regions from being virtually complete information in some regions to very low percentages in several other regions.

Between 1998 and 2017, the educational composition of deaths has substantially changed (Supplementary Table 3). While the lowest educational group has diminished four-fold, the groups of secondary and higher education have increased by about 50%. The percentage of the missing values constituted 6.3% in 1998 and increased to 23.7% in 2017.

**Supplementary Table 3.** Distribution of deaths^a^ by
education in 1998 and 2017 including missing values

|  | 1998 | 2017 | Total |
| --- | --- | --- | --- |
| Unknown | 94677 | 335636 | 430313 |
|  | 6.3% | 23.7% | 14.8% |
| Lower | 836582 | 205384 | 1041966 |
|  | 55.8% | 14.5% | 35.8% |
| Secondary | 470372 | 724481 | 1194853 |
|  | 31.4% | 51.1% | 41.0% |
| Higher | 96506 | 151080 | 247586 |
|  | 6.4% | 10.7% | 8.5% |
| Total | 1498137 | 1416581 | 2914718 |
|  | 100% | 100% | 100% |

*Note*. ^a^ Death records with missing age excluded. Ages 30 to 80.

Section A of Table 1 in the main text presents outcomes of the logistic regression model that uses unknown education as a separate category of education. These results suggest that OR values for the unknown education are lower in 2017 compared to 1998. While in 1998 the odds of non-leukemia death for the unknown education are approximately at the level of the lower education category, in 2017 these odds are close to the level of the secondary education (or approximately the entire national population). This finding reveals an important difference between 1998 and 2017 in the mechanism leading to missing education on death records. In 1998, deaths with unknown education of the deceased originated from a small and selective population that experienced a very high risk of death. This evidence corresponds to prior foreign studies relying on register-based or census-linked data and suggesting that these deaths occurred among people with lower education. ^s2, s3^

In 2017, deaths with unknown education constitute a much larger subset.. The missing education category is to larger extent attributable to administrative shortcomings in some regions. In particular, these shortcomings concern substantial delays in fully reintroducing the field indicating education of the deceased following the corresponding federal decision in 2011. t. Our preliminary analysis (not shown here) demonstrated that the administrative region (*oblast*) was the strongest predictor of missing education in 2017. The share of missing education on death certificates shows a huge variation across regions from a low of 1.2% in Smolensk oblast to a high of 58.1%, 62.5%, and even 100% in Sankt Petersbourg, Sakhalin Oblast, and the Chechen Republic, respectively.

The Multiple Imputation (MI) method assumes missing at random (MAR) data. However, very high mortality rates observed in the missing education category in 1998 and relevant evidence from other countries suggest that the Russian death certificate-based data for this year cannot be regarded as the MAR because there is a likely relationship between propensity of education to be missing and its values. Therefore, we excluded the missing values of education in 1998 from the imputation procedure. The observed mortality rates and the share of missing education suggest a completely different situation in 2017. This difference is attributable to a large variation of the incompleteness of reporting of education across regions. Therefore, we assume that the data of 2017 can be considered as the MAR and use MI to fill in missing values of education in this year.

We carried out the regression-based imputation assuming that several explanatory variables may contribute to a more precise estimation of education for the missing cases. Thus, the multiple imputation model includes variables from the main explanatory model (case-control (leukemia vs non-leukemia) and age group) and auxiliary variables, including sex, urban-rural residence, and region. The auxiliary variables were chosen under the assumption that they are either associated with education or related to the missing-data mechanism.

The last step was running a multiple imputation model by implementing the corresponding algorithm based on multinomial logistic regression in STATA (mi impute mlogit). This solution allowed to fill the missing values in the polytomous education variable on the basis of the selected aforementioned predictors (variables). The number of imputations *M* was set to 30. We also experimented with 10 and 20 imputations and found that *M*≥20 ensures the almost full absence of variation of outcomes.

The estimated distributions by education in the imputed data for each sex, leukemia, and non-leukemia deaths for the year 2017 are shown in Supplementary Table 1. These distributions are similar to those in the complete original data. As one could expect, the share of lower education in the imputed data is higher (by about 5%) and the shares of secondary and lower education are somewhat lower. Finally, we ran on the imputed data the main explanatory (logistic regression) model linking the case-control variable with education and age within each sex. The results are presented in section C) of Table 1 in the main text.

**References**

S1 Kharkova TL, Nikitina SY, Andreev EM. Zavisimostj prodolzhitel’nosti zhizni ot urovnia obrazovanya v Rossii [Dependence of life expectancy on the education levels in Russia]. Voprosy Statistiki 2017;8: 61–69.[in Russian]

S2 Kunst AE. Groenhof F. Potential sources of bias in unlinked cross-sectional studies. In: Kunst AE, eds. Socio-economic inequalities in morbidity and mortality in -Europe: a comparative study. Rotterdam: Erasmus University, 1996: 147–62.

S3 Martikainen, P., Valkonen, T. Bias related to the exclusion of the economically inactive in studies on social class differences in mortality. International Journal of Epidemiology 1999, 5: 899–904

S4 Hardt J., Herke M., Leonhart R. Auxiliary variables in multiple imputation in regression with missing X: a warning against including too many in small sample research. BMC Medical Research Methodology 2012, 12:184. <http://www.biomedcentral.com/1471-2288/12/184>
